# A comprehensive review of radon concentrations and annual effective dose amongst workers in copper mines

**DOI:** 10.1093/rpd/ncaf102

**Published:** 2026-03-29

**Authors:** Elvis Sikapizye, Patrick Hayumbu, Ng’andwe Mumba, Peter-John Jacobs, Isidro A Pérez, M Ángeles García, Phoka C Rathebe

**Affiliations:** Department of Physics, School of Mathematics and Natural Sciences, Copperbelt University, P. O. Box 21692, Jambo Drive, Riverside, Kitwe 10101, Zambia; Department of Chemistry, School of Mathematics and Natural Sciences, Copperbelt University, P. O. Box 21692, Jambo Drive, Riverside, Kitwe 10101, Zambia; Department of Physics, School of Mathematics and Natural Sciences, Copperbelt University, P. O. Box 21692, Jambo Drive, Riverside, Kitwe 10101, Zambia; Department of Occupational Hygiene and Radiation Protection, Sedulitas CC, P. O. Box 101169, Richards Bay, KwaZulu-Natal 3901, South Africa; Department of Applied Physics, Faculty of Sciences, University of Valladolid, Paseo de Belén, 7, 47011 Valladolid, Spain; Department of Applied Physics, Faculty of Sciences, University of Valladolid, Paseo de Belén, 7, 47011 Valladolid, Spain; Department of Environmental Health, Faculty of Health Sciences, University of Johannesburg, Doornfontein Campus, P.O. Box 524, Johannesburg 2006, South Africa

## Abstract

Radon, a carcinogen for lung cancer is chemically inert, colourless, odourless, and a radioactive gas that originates from uranium and thorium radioelements. This review article presents radon concentrations and annual effective dose (E_y_) amongst workers in copper mines, as well as examining factors influencing radon levels. Findings indicate varying radon concentration levels ranging from acceptable values to those exceeding the occupational exposure limit of 1000 Bq.m^−3^. For instance, mean radon concentrations ranged from 6.6 Bq.m^−3^ to 2400 Bq.m^−3^ under mechanical ventilation, increasing by a factor of eight under nonmechanical ventilation. Similarly, E_y_ values ranged from 0.80 mSv.y^−1^ to above the 20 mSv.y^−1^ threshold, reaching as high as 34 mSv.y^−1^. Ventilation emerged as the prominent factor influencing radon concentration, alongside temperature, seasonal changes, and mining activities. The study recommends adequate ventilation and routine radon monitoring to improve air quality and protect workers.

## Introduction

The mining industry plays a vital role in the world economy. Despite the economic benefits that mining activities offer to many countries across the globe, excavating mineral ores and processing them may present health hazards such as radon gas [1–3]. Unlike the generally low concentrations of radon found in dwellings with significant air exchanges with the outdoor environment [4, 5], an underground mine drift constitutes a confined workplace that can maintain elevated levels of radon concentrations [6–8]. This health risk has been recognised since the 15th century, when a German physician called Georgius Agricola observed high mortality amongst miners due to a respiratory disease [9], which was later identified to be lung cancer [10]. The association between the observed respiratory disease and radon was linked to the discovery of the properties of radioactive substances in the 20th century, which identified radon as the cause of lung cancer amongst miners [11]. Furthermore, epidemiological studies conducted on cohorts of underground miners provided additional evidence of radon’s impact on human health [12, 13].

Radon is chemically inert, colourless, odourless, and a radioactive gas that originates from the decay of naturally occurring radioactive materials, specifically those containing uranium and thorium radioelements [14, 15]. Their primordial radionuclides have natural abundances of 99.275%, 0.720%, and 100% for ^238^U, ^235^U, and ^232^Th, respectively [16, 17]. The isotopes ^235^U, ^238^U, and ^232^Th all produce radon isotopes, namely actinon ^219^Rn (t_1/2_ of 3.96 s), radon ^222^Rn (t_1/2_ of 3.82 days) and thoron ^220^Rn (t_1/2_ of 55.60 ), respectively [16, 18]. Studies on radium conducted by Curie *et al.*, Debierne, and Schmidt also identified radon as a radioactive gas and led to the isolation of radon isotopes [19–21]. The contribution to radioactive hazards by ^220^Rn and ^219^Rn is relatively low owing to their short half-lives [22, 23], whereas due to its considerably high half-life, the ^222^Rn isotope, coupled with high natural abundance of ^238^U, is considered a major radiological hazard [24]. Radon (^222^Rn) decays with emissions of $\alpha$ particles and four short-lived progeny, namely ^218^Po, ^214^Pb, ^214^Bi, and ^214^Po [12, 22]. Amongst the four radon progeny, the alpha-emitting isotopes, ^218^Po and ^214^Po, are identified as the main carcinogenic agents responsible for lung cancer [25–28], emitting significant alpha particles with energies of 6.00 MeV and 7.69 MeV, respectively [29, 30]. Despite the lung dose primarily caused by the alpha-emitting radon progeny rather than radon gas, the use of radon concentration levels is considered appropriate for estimating the risk of lung cancer [31].

Radon, a gas that can permeate rocks, cracks, holes, and soil is ubiquitous in the lithosphere due to traces of uranium found in nearly all types of rock and soil, with varying concentrations in specific sites and geological compositions [8, 32]. In a mining environment, radon gas comes from different sources, such as backfill mill tailings, ore body, mine water, and broken ores [33–36]. The process of radon exhalation from rock surface into the mine atmosphere starts with the emanation of its atoms from the solid ore-grain. Not all the radon atoms that decay from radium bearing ore makes it into the atmosphere because a substantial fraction remains trapped within the rock. According to Sakoda *et al.* grain size of more than 10 μm in diameter rarely emanates radon atoms [37]. As a result, only radium atoms within the vicinity of the recoil range from the surface of the ore grain can produce radon atoms that can be emanated. This causes radon decay atoms to have a low emanation fraction, which is attributed to the short recoil range of $\alpha$ atoms in solids, typically a few tens of nanometers. The emanated radon is driven towards the surface by diffusion and/or advection through interstitial spaces between the solid grains, and is finally exhaled into the mine atmosphere [38, 39]. Furthermore, radon emanation shows a direct correlation with increasing temperatures [40]. The influence of a rise in temperature can be explained by the reduction in adsorption of radon atoms into grains that occurs during the diffusion process through the porous material [41]. Moisture content is another factor known to influence the movement of radon gas. Increased radon emanation due to moisture content is attributed to increased stopping power for water compared to air, minimising the loss of recoil radon atoms that might otherwise be trapped in adjacent grains [38, 39]. Though a direct proportionality to moisture content is observed only within a specific range, beyond which, any further increase results in reduced radon exhalation [41, 42]. In addition to moisture content, factors such as lithological mineralisation, radium content, and ore grade play a crucial role in influencing the emission of radon gas [35, 43–46]. Radon is moderately soluble in water [27, 47], and dissolves as it percolates and seeps through the micropores of the uraniferous strata. The percolation of water is enhanced by the presence of high pressure within the interstitial pore spaces that facilitates the process and seepage of water in the mine strata. The overall movement of radon into a wet mine may even be more than that of the dry mine because the flow rate of water from the interstitial pore spaces of the material into the mine may be more rapid than the advective flow and the diffusion processes that occur in dry materials [34, 48].

The exposure to short-lived radon progeny puts the sensitive bronchial epithelial cells of living tissues at risk, subjecting them to ionising radiation in the form of $\alpha$ particles [25]. The interaction of $\alpha$ particles with living cells affects the normal biological functions and structure of living tissues [49], and is capable of ejecting electrons from the orbital shells of an atom [50]. This action results in the transfer of energy to the living tissues, leading to absorption and potential damage to the cells [49]. Consequently, this culminates in the mutation of the deoxyribonucleic acid of epithelial cells in the respiratory tract, resulting in the development of lung cancer [2, 51, 52]. Several cohort and case-control studies have been carried out to evaluate the risk of lung cancer linked to radon exposure in occupational and residential settings. These studies have consistently demonstrated a statistically significant positive correlation between radon exposure in mining environments and an increased risk of lung cancer [25]. Radon has been found to be a cause of deaths in different parts of the world. For instance, around 1100 people are estimated to lose their lives annually in the UK due to exposure to ^222^Rn and ^220^Rn [53], while about 300 cases of lung cancer were recorded annually in dwellings in Ireland [54]. Furthermore, a project carried out in the United States to assess the risk of lung cancer amongst the public exposed to residential radon determined that 10% –15% of all lung cancer deaths could be linked to radon exposure [55]. The International Agency for Research on Cancer classified radon as a group 1 carcinogenic agent [56], while the World Health Organization reported radon to have caused more than 15% of lung cancer worldwide, and rated it as the primary cause of lung cancer amongst non-smokers and the second leading cause overall, after smoking [2]. It is for this reason that the International Atomic Energy Agency set the occupational exposure limit (OEL) for radon concentration at 1000 Bq.m^−3^, while the International Commission on Radiological Protection maintained the annual effective dose (E_y_) for occupational workers at 20 mSv.y^−1^ [51, 57]. For this reason, 1000 Bq.m^−3^ and 20 mSv.y^−1^ will be used as reference values for OEL and E_y_, respectively.

In view of the ubiquitous nature of radon and the health effects that have been linked to it, this review article aims to establish a general profile of radon concentrations and E_y_ amongst workers in copper mines. The study also considered factors that influence the concentration of radon gas in copper mines. The article can contribute to the work of policymakers, regulatory bodies on radiation exposure as well as occupational safety and health practitioners in mitigating radon exposure in copper mines.

## Methods

**Figure 1:**
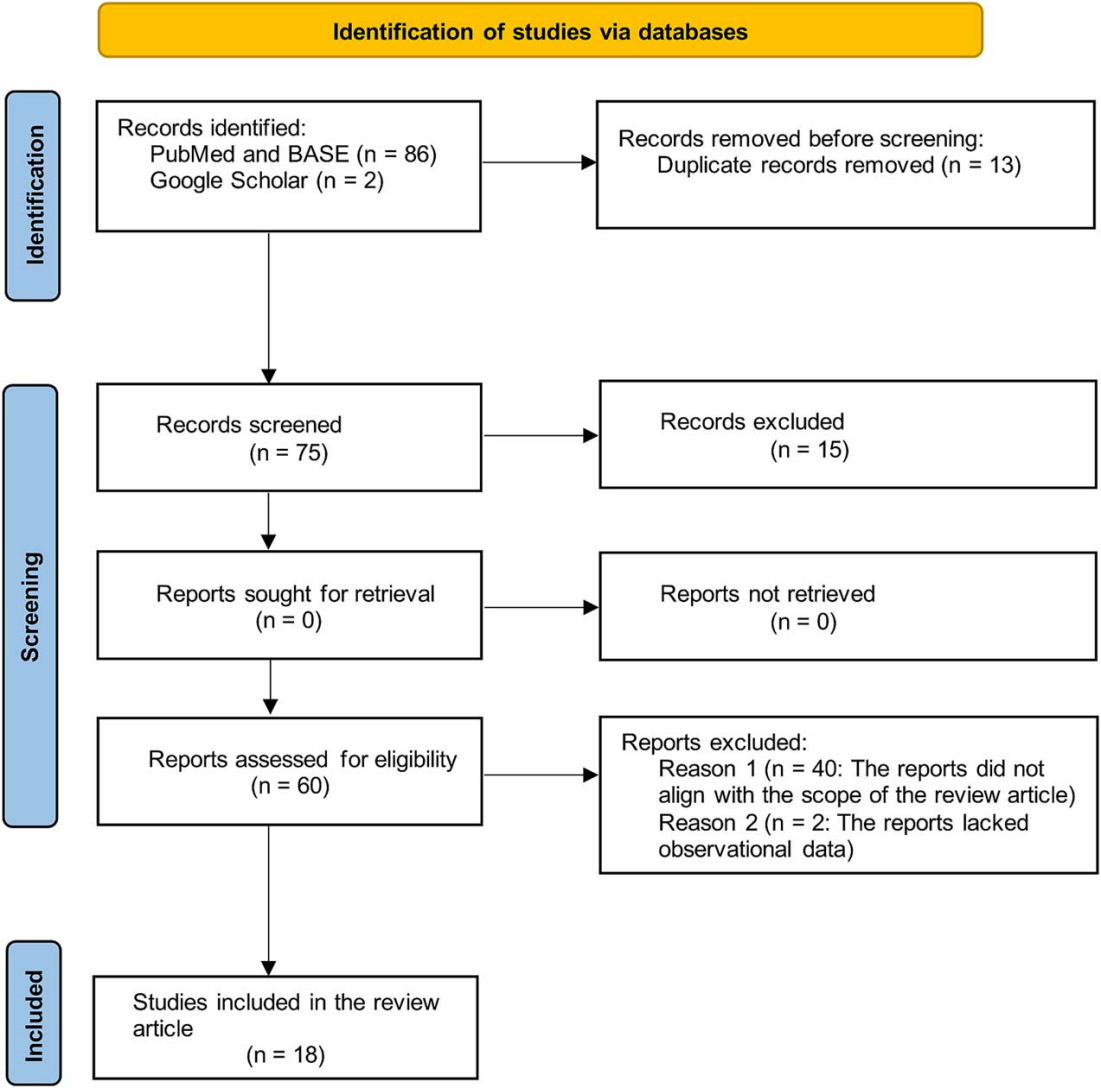
Flow diagram depicting the selection of the 18 articles under review.

The review examined studies that utilised both active and passive characterisation techniques in measuring radon concentration in underground and open-pit copper mines. It included radon monitoring in both active and inactive copper mines. Inactive mines were included due to the fact that such mines are usually considered for modern mineral explorations as well as a source of revenue through tourism when used as tourist attraction sites [27]. But, suffice it to say the main emphasis was on active copper mines. The study considered relevant peer-reviewed journal articles from a wide range of scientific databases, institutional reports as well as publications at meetings such as symposia, conferences, or congresses. A search in PubMed and BASE databases using search terms ‘radon, dosimetry, copper mine, radioactivity’ when executed gave a total of 86 results, out of which 13 were duplicates. Google Scholar yielded two articles that were included. A variety of abstracts were assessed to determine their relevance to the subject matter by Messrs Sikapizye and Hayumbu, and by consensus narrowed down to 18 for inclusion. The review was limited to 18 articles due to the current knowledge gap in the monitoring of radon in copper mines. Figure 1 depicts the flow diagram used in selecting the articles under review generated using Shiny App [58].

## Results

### Synthesis of results

The objective of this study was to determine a general profile of radon concentrations, and to assess the potential risk to human health through the determination of E_y_ amongst miners in workplaces where copper is primarily sought. In this study, radon concentration values are reported to two significant figures due to inherent systematic error in measurements, which results in an analytical accuracy of ~10% relative to the true value [59]. The study also analysed the factors that influence the concentration of radon gas in copper mines.

Table 1 shows data synthesised from the 18 articles included in this review. The trend in the measuring techniques has shown a reduction in the use of working level monitors, passive fission track detectors (PFTDs) and Lucas cells compared to contemporary methods employing mainly the passive track detectors (PTDs) in the form of CR-39 and active methods in the form of AlphaGUARD and RAD7.

**Table 1:** Radon concentrations, E_y_ and the general observations in copper mines.

Author (Year) Country	Detection method	No. of mines	Radon concentration (Bq m^−3^)				Dose (mSv y^−1^)			General observation(s) on radon gas
			Min	AM	Med	Max	Min	AM	Max	
Domanski *et al.* (1981) Poland	- PTD	1	_	_	2000	_	_	_	_	- Properly calibrated PTDs could be used in place of air sampling technique in determining radiation hazards.
	- Air Sampling		_	_	1900	_	_	_	_	
Ahlman *et al.* (1991) Finland	Lucas Cells	2	_	1900	_	19 000	_	_	_	- Attributed the observed slight excess of lung cancer mortalities to exposure to radon daughter products.
Singh *et al.* (1999) India	PTD using ‘sealed can technique’	1	12 000	14 000	_	17 000	_	_	_	- Observed a positive correlation between radon exhalation and the concentration of uranium. - Suggested the need to have adequate ventilation system to remove radon and its associated progeny.
Sengupta *et al.* (2001) India	PTD using ‘sealed can technique’	1st	2700	11 000	_	23 000	_	_	_	- High radon emanation from the rocks and soil. - Suggested the implementation of appropriate controls as well as the monitoring of radon on a regular basis.
		2nd	17 000	21 000	_	28 000	_	_	_	
Evangelista *et al.* (2002) Brazil	Electrostatic precipitation	1	15	150 & 1200	_	2400	_	0.80	20	- Ventilation and mining activities like blasting and drilling plays a pivotal role in the concentration of radon. - Emphasised the need for real-time techniques. -19,000 Bq m^−3^ was obtained while the ventilation system was off, and 2400 Bq m^−3^ was obtained with ventilation system switched on, but with blasting and drilling activities.
Kumar *et al.* (2003) India	PTD using ‘sealed can technique’	1st		11 000	_	_	_	_	_	Observed a positive correlation between radon exhalation and the concentration of uranium.
	PTD using ‘sealed can technique’	2nd	1100	2300	_	4100	_	_	_	
Grattan *et al.* (2004) Jordan	PFTD (1st Survey)	1st	540	630	_	740	_	17	_	- Ancient workers could have been exposed to a greater health risk due to radon gas. - Observed variations in radon concentration due to changes in seasons.
	PFTD (2nd Survey)		660	810	_	980				
	PFTD (1st Survey)	2nd	410	670	_	1000	_	20	58	
	PFTD (2nd Survey)		250	1000	_	2500				
	PFTDs (2nd Survey)	3rd	260	360	_	500	_	8.4	_	
Hayumbu *et al.* (2005) Zambia	- Pylon working level -Lucas Cells -PTD	1	_	_	_	_	6.5	16	35	- Overestimation of exposure dose derived from environmental measurements when compared to dose determined by personal samplers.
Kisolo *et al.* (2007) Uganda	Radon gas monitor	1	330	480 (level 4050) 5300.0 (level 4600)	_	7000	_	2.1	34	Elevated radon levels attributed to poor ventilation, and the movement of radon through cracks and water.
Chau *et al.* (2008) Poland	PTD	4	_	_	_	_	_	0.90	_	- Generally low radiological hazards. - Instances of elevated levels of radon attributed to poor ventilation and turbulent flow of water.
Kisiel *et al.* (2010) Poland	AlphaGUARD	1	12	28	_	49	_	_	_	Low background radiation.
Gillmore *et al.* (2011) United Kingdom	PTD	1	80	610		2200	_	_	_	Archaeologists who visit the abandoned mines could be exposed to the risk of elevated concentrations of radon gas.
Gruber *et al.* (2014) Austria	- AlphaGUARD -EQF 3120 - RTM 2100 - RADIM	1	_	4900	_	_	_	_	16	- Observed the dependence of radon concentration on seasonal changes, with summer registering maximum count. - The survey noted elevated mean radon concentrations in copper mines amongst other non-uranium mines.
Fan *et al.* (2016) China	PTD	1	450	1600	_	1900	_	11	_	- Exposure to workers in production or drifting section was higher compared to other job types like drivers and safety inspectors. - The use of environmental PTDs to estimate dose is likely to result in overestimation. - Variations in radon concentration were observed in different seasons. - Recommended routine personal monitoring of radon exposure.
Polaczek-Grelik *et al.* (2016) Poland	RAD7	_	15	_	_	_	_	_	_	Low background radiation.
Sun *et al.* (2020) China	PTD	1	590	4500	_	19 000	_	18	_	Elevated radon concentration requiring remedial actions.
Szkliniarz *et al.* (2021) Poland	RAD7	1	0.60	6.6	4.8	20	_	_	_	Low background radiation.
Nguyen *et al.* (2022) Vietnam	_	1	16	48	_	480	_	2.3	_	Workers developed respiratory, genetic, digestive, neurological and urological related illnesses.

### Radon concentration and annual effective dose (E_y_)

**Figure 2:**
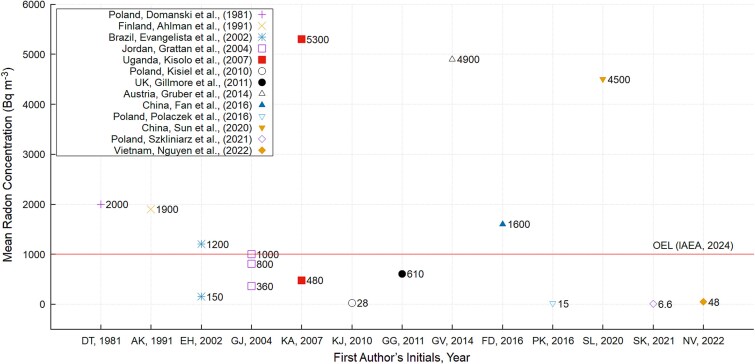
Mean environmental radon concentrations in selected copper mines.

Figure 2 shows the general profile of aggregated mean environmental radon concentrations for selected copper mines ranging from levels deemed acceptable to those greater than the OEL in both active and inactive mines. It shows that seven articles reported elevated radon concentrations exceeding the OEL, while one article reported a radon concentration equal to the reference level of 1000 Bq.m^−3^, which serves as the action level requiring remedial interventions aimed at reducing concentrations of radon gas.

**Figure 3:**
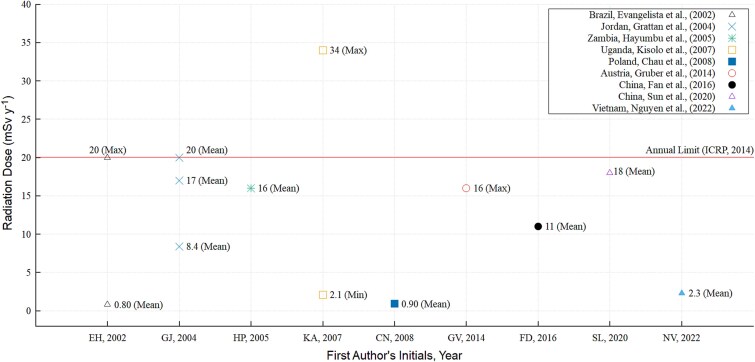
Annual effective dose estimations from environmental radon measurements for miners in selected underground copper mines.

The least mean concentration value of 6.6 Bq.m^−3^ was reported in a study conducted at the Kombinat Górniczo–Hutniczy Miedzi, a deep Polish underground copper mine which aimed at assessing, amongst other objectives, the assessment of natural radioactivity that included the concentration of radon gas in order to establish an underground laboratory [60–62]. The maximum mean concentration was reported by Kisolo *et al.* to be 5300 Bq.m^−3^ [6]. Similarly, Fig. 3 profiles the E_y_ for mine workers in selected copper mines, estimated from environmental area detection techniques. The values ranged from acceptable levels to those exceeding the 20 mSv.y^−1^ threshold. Exceeding the E_y_ limit serves as a crucial indicator that should prompt the need to carry out remedial actions aimed at reducing the concentration to acceptable levels [30, 63]. Figure 3 shows that three articles reported estimations that matched or exceeded the E_y_ dose limit.

The radon measurements in copper mines reported by Hayumbu *et al.* involved two data sets, namely, a first data set collected in 1998 during the preliminary survey of environmental radon measurements at eight different copper mines and a second data set of the follow-up study done in 2004 at a copper mine, which had the most radon concentration values above a worker’s radon OEL during the preliminary survey [64]. The follow-up study collected both environmental radon concentration data and personal radon dose data. Evaluation of these two data sets was done by comparing miners’ workplace radon exposure concentration values against the radon OEL and comparing miners’ radon exposure dose values against the radon annual exposure dose limit. The results of the follow-up personal radon dose study of 2004 showed a huge discrepancy in the E_y_ for environmental concentrations and personal dosimeters. Specifically, the study recorded 6.5, 16, and 35 mSv.y^−1^ against 0.80, 2.6, and 5.9 mSv.y^−1^ as the minimum, mean and maximum dose from environmental concentrations and personal dosimeters, respectively.

Articles that employed radiometric studies generally reported elevated radon activity concentrations. These were not included in Fig. 2 and Fig. 3 because such studies investigated radon exhaled and concentrated in metal cans, rather than in ambient working areas for copper miners [7, 8, 65].

### Factors affecting radon concentration

#### Ventilation

The effect of the ventilation system on radon concentration can be effectively elucidated by first observing radon levels in both active and inactive mines. This is because most inactive mines, or those placed under care and maintenance have mostly relied on natural ventilation, and rarely run the mines under a continuous mechanical ventilation system. This includes a dosimetric study conducted in Uganda’s Kilembe Underground Mine which recorded area concentration levels ranging from 330 to 7000 Bq.m^−3^, with a mean value of 5300 Bq.m^−3^ and an E_y_ of 34 mSv.y^−1^ [6]. Further insights from an Austrian copper mine revealed a mean annual radon concentration of 4900 Bq.m^−3^, with E_y_ for the underground copper mine workers estimated at 16 mSv.y^−1^ [66]. A study conducted in Brazil that investigated the effect of ventilation on radon concentration in an active underground copper mine, observed values ranging from 15 Bq.m^−3^ with a mean of 150 Bq.m^−3^ and an E_y_ of 0.80 mSv.y^−1^ under ventilated conditions. Under nonventilated conditions, a spike to 19 000 Bq.m^−3^ and an average of 4400 Bq.m^−3^ were observed [67]. The discrepancy in E_y_ between estimations derived from personal monitors and environmental monitors is mainly attributed to the ventilation system. For instance, based on the annual averages of ^222^Rn concentrations monitored in Chinese underground copper mines by Fan *et al.*, E_y_ was estimated to be 6.6 mSv.y^−1^ and 11 mSv.y^−1^ for personal and environmental monitoring, respectively. In addition, Hayumbu *et al.* reported an overestimation of E_y_ by a factor of four [64, 68].

#### Temperature and seasonal changes

Some copper mines have associated the variations in radon concentration with temperature changes that arise due to different seasons [27, 69]. The influence of temperature is mostly observed amongst workers experiencing higher radon exposure during summer and the lowest during winter [66]. For instance, Fan *et al.* recorded environmental radon exposure ranging from 480–1900 Bq.m^−3^, 550–2500 Bq.m^−3^, 470–1800 Bq.m^−3^, 330–1400 Bq.m^−3^ during spring, summer, autumn, and winter, respectively [68].

#### Mine type, mining activities, and type of work

Both underground and open-pit types of mines are used in extracting copper ores. In this review, only the underground mines recorded radon concentrations above the OEL [6, 32]. It is observed that the concentration of radon gas and E_y_ also differ within the same mine based on the mining activities being undertaken, and the type of work that different mine workers perform. A Brazilian copper mine that reported a mean value of 150 Bq.m^−3^ observed peaks as high as 2400 Bq.m^−3^ in radon concentration with surges following drilling and blasting activities [67]. An underground copper mine in China reported radon exposure ranging from 630–1700 Bq.m^−3^, 270–650 Bq.m^−3^, and 62–140 Bq.m^−3^ for mining/drifting workers, drivers, and safety inspectors, respectively [68]. In addition, a study conducted in Vietnam reported a huge discrepancy in radon concentration based on the type of work, where offices in non-production areas recorded as low as 35 Bq.m^−3^, while mining pits recorded as high as 430 Bq.m^−3^ [32].

## Discussion

The study analysed articles on radon concentrations and E_y_ for workers in both open-pit and underground copper mines. It was observed that underground mines generally record higher concentrations of radon and its progeny compared to open-pit mines due to their confined nature allowing the accumulation of the radioactive gas as opposed to open-pit mine excavations that allow continuous air exchange with atmosphere [6, 32]. The study has also shown varying results, ranging from acceptable area radon concentrations and E_y_ to those above the recommended limit of 1000 Bq.m^−3^ and 20 mSv.y^−1^, respectively [51, 57]. The mean radon concentrations and E_y_ varied from 6.6 Bq.m^−3^ to 5300 Bq.m^−3^ and 0.80 to 34 mSv.y^−1^, respectively.

The study has demonstrated inhomogeneous distribution of radon concentration within the same mine, owing to the higher dispersion of measurements from the lowest to highest values. In some instances, the highest value being more than four times the lowest [6, 32, 68]. For instance, Evangelista *et al.* observed radon concentration to over four times the baseline levels under a steady ventilation system [67]. This phenomenon may be attributed to, amongst other reasons, differences in the distribution of ^226^Ra and the internal structure of the source material, affecting radon emanation from the grain to the pore spaces [70]. Furthermore, variations in radon concentrations are equally influenced by mining activities involved in the extraction of metals such as drilling and detonations that result in increased radon exhalation from rock fracturing [67, 71]. The enhanced emanation of radon from rock fracturing is due to increased exposure to new rock surfaces containing traces of uranium and resulting in the radon trapped within the matrix of the rock escaping and contribute to the concentration in the mine atmosphere [72]. In addition, these mining activities cause surge events that result in sudden increases in radon concentration [67]. Seasonal changes also have a profound influence on variations in radon concentrations, in some instances causing changes by a factor of 10 [27]. Specifically, meteorological factors like humidity, pressure, and temperature differences between the underground mine and the surface cause variations in radon concentrations [66, 69]. It is observed that summer and winter depict changes in radon concentrations with the former registering higher levels of radon compared to the latter [27, 66, 68]. This is attributed to higher summer temperatures enhancing the exhalation of radon from rocks, with lower air pressure allowing the gas to accumulate more easily [40, 73]. According to Arrhenius Theory, a temperature increase of several degrees Celsius will result in easy radon escape out of the grains because of increased radon diffusion coefficient in solid materials [74, 75]. In winter, the warm air within the mine rises toward the atmosphere, carrying away radon and its progeny, resulting in reduced radon concentration in the mine [76]. Moreover, high humidity levels influence the concentration of radon progeny due to enhanced attachment of progeny to aerosol particles resulting in an increased equilibrium factor [77].

This study has revealed instances of radon concentration measurements in copper mines exceeding the OEL [6, 27, 64, 66–68, 78]. At least 11 out of the 18 articles considered have attributed the effect of radon concentration to the ventilation system, signifying the significant role that it plays in regulating the concentration of the radioactive gas and its progeny [6–8, 27, 65–68, 79]. For instance, Kisolo *et al.* attributed the high radon concentration to poor ventilation despite rock samples recording low mean activity concentration of thorium and uranium [6]. The effect of ventilation is more pronounced in abandoned mines, yet such mines, which attract tourists and archaeologists should be treated with caution because of the lack of consistent mechanical ventilation which may pose a risk of exposure to accumulated radon gas [66, 69]. Typically, ancient copper mines had very poor ventilation systems which potentially exposed mine workers to a significant health risk [27, 78]. Over time, construction of mine shafts and installation of mechanical ventilation systems have resulted in improved air exchange between the inside and outside of the mine. It is for this reason that the most effective remedial measure currently being undertaken to reduce elevated radon concentration levels in copper mines is the improvement of the ventilation system [67].

E_y_, which involves the assessment of the estimated dose over a period of one year due to exposure to radon and its progeny, serves as a radiation protection quantity that can be changed as a result of new knowledge [51]. We observed overestimation of E_y_ when comparing environmental and personal radon monitors, an occurrence largely attributed to the effect of ventilation, where the former has registered a higher count compared to the latter [64, 68]. The huge discrepancy in the estimation of E_y_ is due to the fact that environmental radon concentration measurements are a time-weighted average that includes moments when the ventilation system is switched off, as well as the overestimation in working time for environmental radon monitors [64, 68, 79]. However, with stable and continuous ventilation system, the gap in E_y_ by the two detection methods can be reduced extensively as observed by Domanski *et al.* though their environmental detectors were deployed for only 7 days [79]. In this case, the use of personal dosemeters is a more reliable method in estimating E_y_ [64, 68, 80]. Another factor influencing E_y_ is the nature of a miner’s job type, even if they all work for the same mine. This is due to the fact that mining or drifting workers who spend more time underground are more prone to elevated levels of radon concentrations compared with other job types, such as mine inspectors from different work sections that spend less time in underground mining galleries [32, 67, 68]. In addition, E_y_ is influenced by the equilibrium factor (F), a variable incorporated into the equation used to estimate exposure risk. Different surveys that determined F within the same mine have reported a continuum of values ranging from 0.2–0.7 [69, 81, 82]. This implies that the common practice of adopting a standard value for F in a dynamic environment like a copper mine which has many factors that influence radon concentration might have a profound effect on the estimation of E_y_ [83], resulting in increased uncertainties in the estimated risk of exposure. In addition, there is lack of uniformity in the adopted standard F used in copper mines [67, 79]. In view of this, we encourage the incorporation of active electronic monitoring instruments like the AlphaGUARD and RAD7 that have the capacity to determine F at each sampling point in order to obtain a more accurate estimation of E_y_.

Non-uranium mines, which include coal, copper, iron, silver, cobalt, and gold are not categorised as nuclear installations, and therefore do not follow strict licencing and protocols aimed at reducing radiological contamination [67]. In addition, this study has demonstrated that not all copper mines are at risk of exposure to elevated levels of radon and its progeny [27, 60–62, 69, 79]. However, based on the geological setup where a non-uranium mine is located, the state of ventilation system and meteorological factors, such mines can record elevated radon concentrations [7, 8, 65, 76]. For instance, the Mosabani Copper Mine, whose geological composition is associated with uranium mineralisation showed heightened radon exhalation rates in its copper ore tailings [7, 8, 65], suggesting a direct link between radon concentration and the geological composition of the mine’s location. It should be noted that the uranium content that may pose a radiological risk may not be adequate for mining exploration, yet it may be enough to cause a radiological health risk to mine workers. Notably, two separate studies that considered radon concentrations in non-uranium mines both found that copper mines registered the highest mean concentrations. The first study surveyed iron-ore, silver, and copper mines, while the second study considered copper, coal, and iron mines [66, 68].

Radon gas can be inhaled during breathing with a substantial amount of it being exhaled, although part of it decays within the respiratory tract [84]. In addition, radon progeny, which exist in the form of charged particles, can attach to dust and natural aerosols and also be deposited into the lungs when inhaled [85]. The deposited radon and progeny are reported to cause lung cancer mortalities and respiratory related infections in some copper mines [32, 78]. A study that considered lung cancer mortalities in a predominantly copper mine township in Finland revealed that 47 out of the 106 people who died from lung cancer were old copper miners. The majority of whom had worked in a copper mine for more than three years and were reported to have been highly exposed to radon progeny and to silica [78]. In Vietnam, a radon survey carried out at the copper mine, coupled with retrospective health data reported an average concentration of 48 Bq.m^−3^, and a maximum of 480 Bq.m^−3^ revealed that at least 45% of mine workers developed lung related diseases [32]. Thus, adverse effects can be experienced even at reasonably acceptable concentrations due to prolonged exposure time, coupled with other synergistic factors experienced by miners such as smoking, diesel particulate matter, respirable crystalline silica, and pre-existing health conditions, all of which contribute to a worker’s exposure [32, 86, 87]. It is observed that the risk of lung cancer due to radon gas is mainly confounded by smoking [78, 88]. Furthermore, Nguyen *et al.* established that the risk of death from lung cancer in males due to exposure to radon increases by a factor of six for smokers compared to nonsmokers [32]. Therefore, it is imperative to populate groups of smokers and nonsmokers when assessing the risk of lung cancer in order to establish the degree of confounding effect caused by smoking [89].

Radon concentrations in underground copper mine galleries exceeding the OEL are a concern for the occupational safety, health and welfare of miners. This article has demonstrated that elevated radon concentrations exceeding the OEL, are not confined to uranium mines alone. Rather, depending on factors such as the ventilation efficiency, temperature, atmospheric pressure, humidity conditions, and geological composition of the mine, elevated levels may also occur in a copper mine [6–8, 65, 67]. It is encouraging to note that some regions of the world have implemented policy directives regarding acceptable radon concentration limits in workplaces and in dwellings. The Council of the European Union directed that EU member countries implement laws related to customised radon occurrence limits in residential buildings and workplaces [90]. Much as the efforts to set exposure limits in workplaces are commendable, it should be noted that radon is a carcinogen for lung cancer with no established safe threshold. This implies that the task should not just end at having concentrations below the OEL, but that the concentration of radon gas should be decreased to as low as reasonably achievable [91].

Considering that some active copper mines have recorded concentrations above the OEL, attention should also be directed towards the monitoring mechanism in the copper-producing mines in order to curb any sudden upward surge in the concentration of radon and its progeny, ultimately enhancing the occupational safety of mine workers. In addition to improved ventilation, areas that are consistently found with elevated levels of radon gas should be declared as controlled places with limited access, and workers of such stations should be mandated to wear personal radon monitors and undergo annual health checks [69]. Furthermore, the mines should consider limiting the amount of time spent in a particular site that consistently records heightened radon concentration in order to ensure that the E_y_ limit is not exceeded because heightened levels of radon exposure can predispose mine workers to the risk of lung cancer [69, 78]. Additionally, it is recommended that lung cancer incidences be established in areas that consistently record heightened radon concentration levels in order to determine the radiological risk to miners [6]. We encourage the use of both passive and active detection techniques in radon-prone underground copper mines in order to enhance radiological protection. Radon detectors such as PTDs are recommended for long-term monitoring, while active instruments are suitable for periodic real-time measurements in monitoring radon dynamics in relatively short time intervals, such as during operational hours [67, 68, 92]. The radon monitoring mechanism is crucial, considering that radon is colourless, tasteless, and odourless [14], making it difficult for miners working in mine galleries to notice by sight or smell any surge in concentration. In addition, the health effects of radon gas and its progeny have a prolonged latency period as opposed to acute illness that can trigger a quick response to ameliorate a hazardous environment [32, 68, 78].

## Conclusion

The study revealed mean radon concentration levels as low as 6.6 Bq.m^−3^ and as high as 2400 Bq.m^−3^ under ventilated conditions, and as high as 19 000 Bq.m^−3^ under nonventilated conditions. Similarly, the E_y_ ranged from 0.80 mSv.y^−1^ to above the 20 mSv.y^−1^ limit, reaching as high as 34 mSv.y^−1^. Based on the observed radon concentration surges in copper mines, this study concludes that routine radon monitoring is essential for underground copper mines to safeguard workers from potentially elevated levels of radon gas and its progeny. As highlighted, ventilation plays a crucial role in regulating radon concentration levels in copper mines. Improved ventilation, with increased air exchange, is considered one of the most effective remedial measures that can be undertaken to reduce the residence time of radon and its progeny in confined workspaces like underground mines. Besides improved ventilation systems in mines that record heightened radon levels, personal dosimeters should be used by workers for more accurate estimation of E_y_. Mine areas with inadequate ventilation that consistently record elevated levels of radon gas should be declared as controlled places with limited access, and workers of such areas should be mandated to wear personal radon monitors and undergo annual health checks. Despite ventilation being the most significant factor affecting radon concentration, temperature, pressure, humidity, and mean activity concentration of primordial radionuclides also play a crucial role on the concentration of the radioactive gas. In addition, the miner’s job type and mining activities also affect the level of radon in a mining environment. Based on the observation of elevated levels of radon concentration above the OEL, coupled with limited literature on radon dosimetry in copper mines, it may be suggested that there is a lack of routine monitoring of radon gas leading to inadequate information about this potential health hazard to workers. In this regard, the monitoring of radon gas on a regular basis can serve as an indicator of the air quality in a mine.

We recommend that copper mines ensure an adequate ventilation system in order to safeguard workers from potential radiation hazards, such as radon gas and its progeny. They should also implement routine monitoring of radon gas and its progeny.
